# Litter and perch type matter already from the start: exploring preferences and perch balance in laying hen chicks

**DOI:** 10.1016/j.psj.2020.11.041

**Published:** 2020-11-27

**Authors:** Lena Skånberg, Cecilie Bramgaard Kjærsgaard Nielsen,, Linda J. Keeling

**Affiliations:** Department of Animal Environment and Health, Swedish University of Agricultural Sciences, SE-750 07 Uppsala, Sweden

**Keywords:** laying hen chick, behavioral preference, litter, perch balance, perch design

## Abstract

Early chick environment, such as provision of litter and perches, can be a predictor of laying hen welfare. Inadequate or nonpreferred litter and perch types could have similar negative effects as those seen when not providing these resources at an early stage, such as increased feather pecking and cannibalism in adult flocks. However, suitable litter and perch types for chicks are not well explored. In the present project, 6 different types of litter (crushed straw pellets, hemp shavings, peat, sand, straw, wood shavings) and 6 different types of perches (narrow or wide forms of rope, flat or round wood) were presented in a controlled way (3 at a time) to chicks in 6 pens. Usage was compared in 93 chicks of Lohmann Selected Leghorn Classic divided across the pens, during their first 3 wk after hatch. Different litter types were seen to be preferred for different behaviors. The majority of dustbathing bouts occurred in sand and peat. Chicks foraged more in wood shavings, hemp shavings, and sand than in peat and pellets (*P* < 0.05). Perch width and shape were found to affect both usage and perch balance, measured as the probability of successful or problematic landings. The wide rope was generally used more during the first week (*P* < 0.05) and was used more for sleeping or resting (*P* < 0.05) than the other wide perch types. Furthermore, birds were more likely to land on the wide rope or on flat perches successfully than they were to have a problematic landing (*P* < 0.05). That birds were more likely to be observed preening on flat perches than on the potentially shaky rope perches could further reflect a sense of security. Our results suggest that presenting several litter types could better fulfill chicks' behavioral needs and that flat perches or a wide rope (4.5-cm diameter) could be appropriate perch types for laying hen chicks and thereby promote early perch use and training.

## Introduction

An appropriate rearing environment is essential for laying hen welfare (Janczak and Riber, 2015). Welfare scores for young flocks can be directly linked to welfare scores for the adult flock. One example is the connection found between higher levels of severe feather pecking in young birds and an increased risk of feather damage as adults (de Haas et al., 2014a). Several factors in the environment for chicks have been identified as potential predictors for the prevalence of various welfare problems in the adult flock. Some risk factors frequently identified are limited access to litter (Gilani et al., 2013) and perches (Gunnarsson et al., 1999). As per EU directives (1999/74/EC), laying hens must be provided access to litter and perches, whereas there is no equivalent requirement for birds during rearing.

Rearing chicks without continuous litter provision could have long-term negative welfare effects (Johnsen et al., 1998; Aerni et al., 2005). Adult birds that were reared without access to litter during the first 4 wk have been shown to have poorer plumage scores, lower egg production, and higher mortality and feather pecking rates along with higher levels of fear (Johnsen et al., 1998; Aerni et al., 2005). Rearing flocks with a 1-week disruption of litter provision (around 4 wk of age) has been found to result in increased levels of severe feather pecking, higher fear levels, and poorer plumage scores at 10 wk of age (de Haas et al., 2014b). However, letting paper and feed function as a litter substrate, a practice commonly used during the first weeks in multilevel rearing systems, may not be enough to have a positive impact on feather pecking levels (de Haas et al., 2014b). This emphasizes the importance of choosing an adequate type of litter in the rearing environment. Litter is considered important for essential laying hen behaviors such as foraging (Blokhuis, 1991) and dustbathing (Larsen et al., 2000; Wichman and Keeling, 2009).

Foraging behavior, such as ground scratching and pecking, starts to develop and increase during the first week after hatch (Vestergaard and Baranyiova, 1996). It has been recommended to stimulate foraging behavior early by providing sufficient litter in the rearing environment because of its potentially suppressive effect on the development of feather pecking (Gilani et al., 2013; Rodenburg et al., 2013). Severe outbreaks of feather pecking have been suggested to be a redirected form of foraging behavior because rearing birds on litter, compared with wire mesh, was found to increase foraging and to decrease feather pecking and pecking at objects during rearing (Blokhuis and van der Haar, 1989). However, research comparing foraging behavior in different litter types in chicks is limited.

Dustbathing is an essential behavior for feather maintenance by removing excessive fat lipids (van Liere and Bokma, 1987; Olsson and Keeling, 2005). The behavior is already evident during a chick's first few days of life (Larsen et al., 2000), and preferences for certain litter types have been identified in adult hens (de Jong et al., 2007) and in young individuals (Sanotra et al., 1995; Vestergaard and Baranyiova, 1996; Shields et al., 2004). Having insufficient dustbathing material could, apart from worsen feather condition (van Liere and Bokma, 1987), induce frustration (Wichman and Keeling, 2009) and increase feather pecking (Larsen et al., 2000). Existing research suggests sand (Sanotra et al., 1995; Shields et al., 2004) and peat (Vestergaard and Baranyiova, 1996; de Jong et al., 2007) to be suitable dustbathing substrates.

Regarding perch use, chicks start using perches when they are 1 wk of age (Heikkilä et al., 2006; Riber et al., 2007). Early perch use has been seen to promote both skeletal development (Yan et al., 2014) and muscle growth (Hester et al., 2013). Chicks without early access to perches may develop impaired cognitive spatial skills (Gunnarsson et al., 2000) and have a higher risk to lay floor eggs and perform cloacal cannibalism as adults (Gunnarsson et al., 1999). It can be hypothesized that the provision of an inadequate perch type could have similar negative effects. There are several different aspects to consider when identifying suitable perch types, such as material, color, shape, width, and height (EFSA, 2005; EFSA 2015). From the animal's perspective, the type of perch could affect balance, thermoregulation, the type of grip, and preference and thereby influence perch use (Pickel et al., 2010, 2011; EFSA, 2005; EFSA 2015). There is limited research exploring which perch material, shape, and width could be appropriate for chicks or pullets (EFSA, 2005; EFSA 2015).

More research has been requested with regard to identifying appropriate facilities for foraging and dustbathing (EFSA, 2005; EFSA 2015) along with adequate perch types (EFSA, 2005; EFSA 2015). Previous preference studies for litter types have used experimental test setups, such as preference tests by pushing weighted doors (de Jong et al., 2007), relocating chicks to a novel test cage (Sanotra et al., 1995; Shields et al., 2004), or allowing brief access (maximum: 35 min) to a box with substrates while otherwise being housed on wire (Vestergaard and Baranyiova, 1996), all of which gives birds access to only one substrate at a time. To our knowledge, no previous study has allowed chicks continuous free access to different litter types in their home pen. Investigating more long-term preferences (i.e., in a more straightforward way, and not potentially affected by litter deprivation, training, or neophobia) could give results that better reflect chicks' behavior in commercial practice. Regarding perches, also to our knowledge, there has been no study that compares chicks' usage of different perch types. Research on perch types conducted in adult hens has been summarized by EFSA Panel on Animal Health and Animal Welfare, 2005, EFSA Panel on Animal Health and Animal Welfare, 2015.

The aim of this study was to investigate chicks' preference and usage of litter and perch types when presented with a choice of different types in their home environment. Identifying and using appropriate litter and perch types for chicks in the rearing environment could lead to greater success in the adult environment.

## Materials and methods

The study was approved by the Animal Research Ethics Committee in Uppsala (Dnr 5.8.18-11,549/2017).

### Birds and Housing

**Figure 1:**
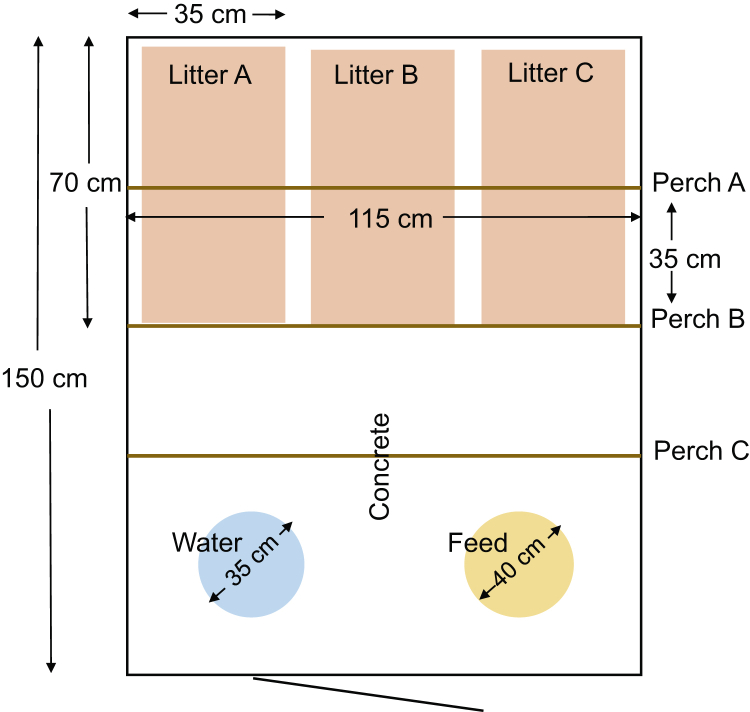
Each pen had 3 different litter types and 3 different perch types at a time, presented at locations A, B, and C. All litter and perch types were presented to all pens and at all locations during the 3-week-long test period.

For the present project, 93 female chicks of the hybrid Lohmann Selected Leghorn Classic, from the same commercial hatchery, were transported 383 km on their first day after hatch to the Swedish Livestock Research Center in Uppsala, Sweden. On arrival, chicks were divided across 6 pens in the same stall: 3 pens with 15 chicks and 3 pens with 16 chicks. The pens (1.15 × 1.5 m) contained feed and water ad libitum (Figure 1). The stall had an initial temperature of 30°C. Heating lamps were placed over the middle of the pens during the chicks' first 5 d. Temperature was then successively decreased to 26°C by day 23. The stall had a fixed night schedule of 6 h of dark (4–10 pm) from day 4. Chicks were given 1 h of dark on day 1 and 2 h of dark on day 2–3 to promote feeding. Before and after each dark period, the room had a twilight period of 30 min when light intensity was continuously reduced or increased. All pens were supervised at least 2 times per day.

### Experimental Setup

**Figure 2:**
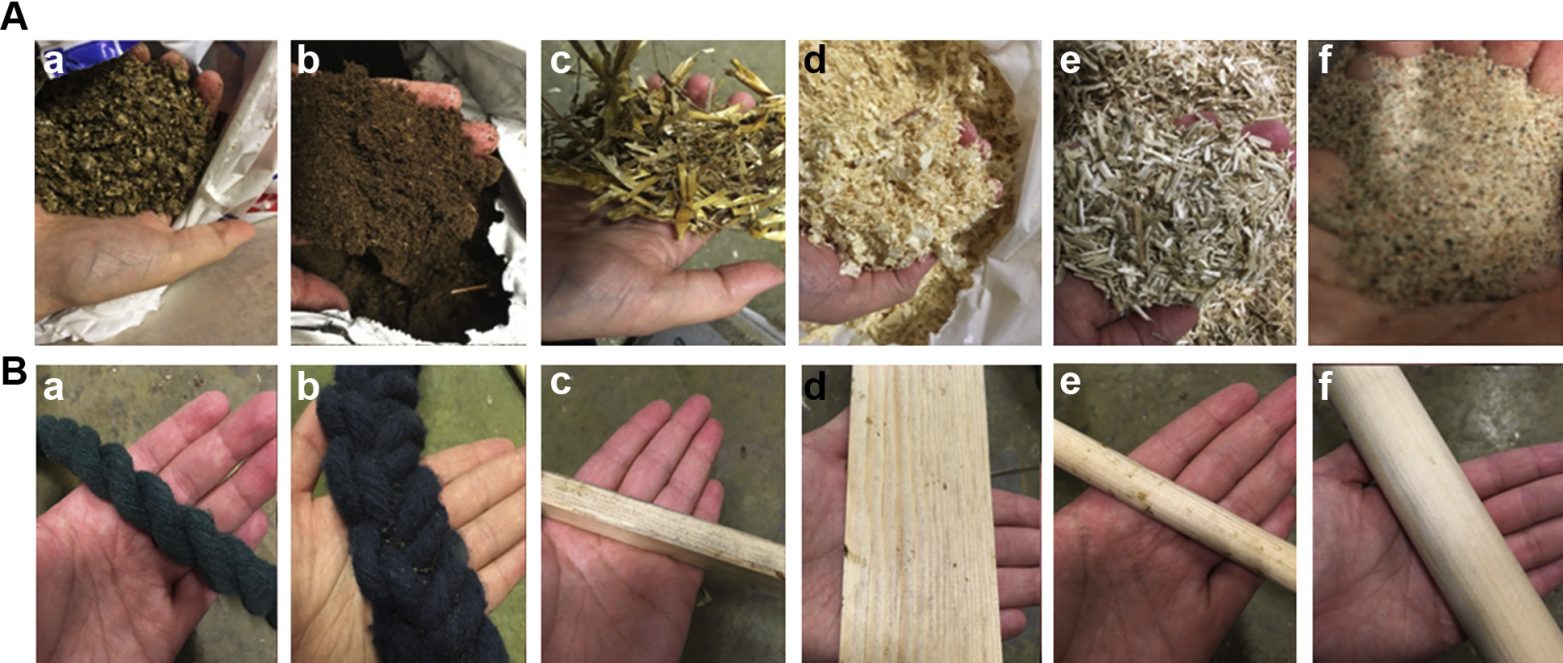
(A) The 6 tested litter types: (a) crushed straw pellets (RS MUSTANG, Enköping, SE), (b) peat (RS MUSTANG, Enköping, SE), (c) straw (Smådjurshalm LUPUS, Granngården Uppsala, SE), (d) wood shavings (Kutterspån GRANNGÅRDEN, Uppsala, SE), (e) hemp shavings (Lättströ GRANNGÅRDEN, Uppsala, SE), and (f) fine-grained sand (0–0.03 mm, RÅDASAND, Djur-Hobby Uppsala, SE). (B) The 6 tested perch types: (a) narrow rope (1.8 cm in diameter), (b) wide rope consisting of 3 narrow ropes braided together (4.5 cm in diameter), (c) narrow flat wood (1.5 × 1.5 cm), (d) wide flat wood (6.7 × 0.8 cm), (e) narrow round wood (1.5 cm in diameter), and (f) wide round wood (3.5 cm in diameter).

The 6 tested litter and perch types are presented in Figure 2. Litter types were presented in trays (70 × 35 × 3 cm) at locations A–C (Figure 1) (one type on each tray). Trays were kept in place by wooden frames in the pens, to which were also mounted 10-cm vertical plastic barriers to prevent litter types becoming mixed. All perches in all pens had the same height. During the first 5 d, all perches were on the ground, to ensure that the chicks came in contact with them. On day 5, we raised the perches to be 15 cm from the ground, which was just enough to allow chicks to pass underneath. When enough birds had started using the perches at a height (at least 3 birds in each pen), all perches were raised a further 5 cm. Perches were raised additionally on day 9, 12, 14, 16, 19, and 21, thereby ending at a height of 45 cm when chicks were 3 wk of age.

Because only 3 litter and 3 perch types could be presented at a time in a pen, the locations of the types were changed within and between pens over the study period so that chicks in all pens came in contact with the different types in a standardized way. This was carried out as per a prepared schedule (Table 1) at midday on Mondays, Wednesdays, and Fridays. By the end of the 3-week period, all litter and perch types had been compared with all others, and each litter type and each perch type had been presented at each of the 3 locations (A, B, and C, Figure 1).

**Table 1:** Standardization procedure.

Period	Pen 1			Pen 2			Pen 3			Pen 4			Pen 5			Pen 6		
	A	B	C	A	B	C	A	B	C	A	B	C	A	B	C	A	B	C
Day 1–5	1	2	3	2	3	4	4	1	5	3	5	6	5	6	2	6	4	1
Day 5–7	4	5	6	5	6	1	6	3	2	2	1	4	1	4	3	3	2	5
Day 7–9	2	4	3	4	5	2	1	6	5	6	3	1	3	2	6	5	1	4
Day 9–12	4	6	1	1	3	5	6	2	4	2	5	3	5	1	2	3	4	6
Day 12–14	3	1	2	5	6	4	2	3	1	1	4	6	4	5	3	6	2	5
Day 14–16	6	5	4	4	1	2	5	4	3	3	2	1	2	6	5	1	3	6
Day 16–19	5	3	1	6	2	3	2	5	6	1	4	5	3	1	4	4	6	2
Day 19–21	1	2	6	3	4	1	5	1	4	4	6	2	6	3	5	2	5	3
Day 21–23	6	4	5	2	1	6	3	6	1	5	2	3	4	5	2	1	3	4

Each of the 6 litter types was randomly paired with one of the 6 perch types, before the start of the experiment, and each pair was assigned a number between 1 and 6. This was to simplify the standardization procedure and decrease risks of errors when changing litter and perch combinations in practice. The table shows which pair of litter and perch type (1–6) was presented at which litter location (A–C) and perch location (A–C, Figure 1) and in which pens (pen 1–6) on which days. Each litter and perch type was presented at all 3 locations (A–C), in different pens at any given time. After the first change (d 5), all pens had come in contact with all litter and perch types.

### Behavioral Observations

Live behavioral observations were divided into 2 parts: “time budget observations,” to determine how chicks were distributing themselves between the different types of each resource and what behavior they were performing in each type, and “perch balance observations,” wherein we observed chicks' abilities to jump up onto and maintain their balance on the different perch types (Table 2). An equal number of observations were made in the morning before the change of litter and perch (i.e., when the birds had access to the resource for at least 36 h) and in the afternoon (within 2 h of the resource change). This was to control for eventual behavioral responses connected to novelty.

**Table 2:** Ethogram over the behaviors observed in the 2 types of observations; “time budget” and “perch balance.”

Behavior	Definition
Time budget	
Sleeping/resting	Sitting or lying down with eyes closed or open
Preening	Chick directs its beak to its own plumage at one of several body parts (thorax, abdomen, shoulder, interior and exterior wings, rump, back and cloaca) and carries out pecking, nibbling, combing or rotating movements once or rapidly. Definition from the study by Pickel et al. (2010).
Foraging	Pecks directed to the floor/substrate while standing or walking and/or the body is bent forward while the bird makes a backward stroke with one leg. Usually 1–4 strokes with one leg are followed by 1–4 strokes with the other. Definition from the study by Blokhuis and van der Haar (1989).
Dustbathing	While lying or squatting, bird performs dustbathing components (bill raking, vertical wing shakes, side lying, rubbing, scratching, ground pecking, feather ruffling). Definition from the study by Newberry et al. (2007).
Moving/standing	The chick is moving or standing and not doing any of the already defined behaviors.
Perch balance	
Successful landing	The landing from the ground on to the perch is stable and without balance-correcting movements.
Problematic landing	The landing involves the body tilting on its axis while tail feathers are spread and rapidly moved up and down, once or repeatedly. The chick's neck may be simultaneously stretched out. Wings are flapped, once or repeatedly, or the chick leaves the perch, without focusing on a landing point, or falls off to the floor. Definition from the study by Pickel et al. (2010).

For “time budget” observations, the behavior of each chick was always noted in combination with its location (litter A–C, perch A–C, concrete, feeder, water).

For “time budget” observations, the location and behavior of each chick was observed by instantaneous scan sampling each pen. See Table 2 for definitions of the different behaviors. We conducted 10 scans per observation day (Monday, Wednesday, and Friday) during 3 wk in each pen during the daytime (5 scans between 9–11 am and 5 scans between 1–3 pm), as well as one observation during the twilight period on these days. At least 15 min passed between each successive scan of a pen to ensure the observations were independent.

For “perch balance” observations, we conducted one 5-min continuous observation per pen on 8 occasions (equally distributed between am and pm) at 5 ages: day 6, 9, 12, 16, and 21 (between 10 am and 3 pm). Observation days were chosen so that the observations were balanced across different perch heights. See Table 2 for definitions of the different behaviors.

A total of 1,764 scans per litter type and per perch type, as well as a total of 720 min of balance observations per perch type, were performed.

### Statistical Analysis

All statistical analyses were performed using R software (version 3.3.2; The R Foundation for Statistical Computing, Vienna, Austria; R Development Core Team, 2016).

Preference or usage of different litter and perch types was investigated using the “time budget observation” scans. For dustbathing, the average number of dustbathing observations in each type of litter is presented because this was a relatively rare behavior. For each of the other behaviors, the average proportion of chicks performing that behavior in each of the different litter types, or on each of the different perch types, is presented. Perch balance was investigated by using the proportions of the total number of landing attempts observed for each pen stratified on landing type (problematic or successful) for each perch type.

All proportions were treated as real values, and linear mixed models were fitted using the restricted maximum likelihood approach using Laplace Approximation and R package lme4 (Comprehensive R Archive Network, Vienna, Austria; Bates et al., 2015).

Litter type preferences were investigated using models with litter types as fixed effects and pen as a random effect. Models investigating perch types used perch width and shape (including a possible interaction) as fixed effects and pen as a random effect. The effect of age (as week) was included in the model for litter or perch type preference if an interaction between age and perch or litter type was found to be significant. An effect of age (as week) was only found for general perch use and sleeping/resting on a perch and was therefore only included in these models. There was no effect of time of observation (hour of the day) on preference, so this factor was left out in the statistical models. For perch balance, the mixed model included perch width, perch shape, and landing type (problematic or successful) as fixed effects (including a possible interaction between them) and pen as a random effect.

Significant fixed effects were investigated using type III ANOVA with Satterthwaite's approximation of degrees of freedom and the lmerTest package (Comprehensive R Archive Network, Vienna, Austria; Kuznetsova et al., 2017). Significant main effects or interactions (*P* < 0.05) were further investigated using pairwise comparisons of the least square means with Satterthwaite's approximation. Pairwise comparisons were adjusted for using Tukey's method.

In the case of heteroscedasticity or a non-normal distribution, which was found for the average number of total dustbathing bouts for each litter type, a Friedman test was used to explore the effect of litter type. Post hoc comparison was made using the Friedman-Nemenyi test with error correction using the Friedman Test Data Analysis Tool for Excel (Microsoft Corporation, WA) in the Real Statistics Resource Pack Software (Release 7.2, Copyright 2013-2020, www.real-statistics.com).

## Results

In the following section, the general time budgets of the chicks are presented, followed by results on how the different litter and perch types were used. Finally, we present how the different perch types affected a chick's ability to land in a stable manner.

### General Time Budget of the Chicks

On average, chicks spent the greatest proportion of their time on the litter (0.5 ± 0.04), followed by being on a perch (0.18 ± 0.04), on the concrete floor (0.17 ± 0.01), at the feeder (0.12 ± 0.01), and at the drinker (0.02 ± 0). Regarding the proportions of observed behaviors, chicks were most frequently foraging (0.43 ± 0.04), followed by sleeping or resting (0.25 ± 0.03), moving or standing (0.22 ± 0.01), preening (0.08 ± 0), and dustbathing (0.01 ± 0.01). Moving/standing was not considered a motivated behavior category, and it is not highly dependent on litter or perch type, so it was not investigated further.

### Litter Type Preferences

**Figure 3:**
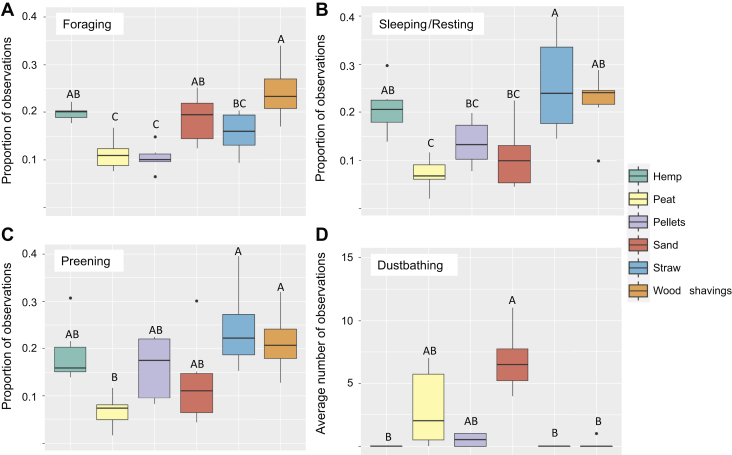
The boxplots show the average proportion of observations when chicks were observed to be (A) foraging, (B) sleeping or resting, or (C) preening in the 6 different types of litter. (D) The average number of observations of dustbathing chicks in the 6 different types of litter. ^A,B^ Different letters indicate significant (*P* ≤ 0.05) differences between litter types. Pairwise comparisons have been corrected for multiple comparisons using Tukey's method.

A main effect of litter type was found in all the investigated behaviors (Figure 3), foraging in litter (F_5,30_ = 9.86, *P* < 0.001), sleeping or resting in litter (F_5,30_ = 7.24, *P* < 0.001), preening in litter (F_5,30_ = 4.59, *P* = 0.003), and the average number of performed dustbathing bouts in litter (χ^2^ = 22.70, *P* < 0.001). In pairwise comparisons, there was more foraging in wood shavings than in pellets (*P* < 0.001), peat (*P* < 0.001), and straw (*P* = 0.01; Figure 3A). There were also more observations of chicks foraging in hemp shavings and sand than in pellets (*P* = 0.006, *P* = 0.02) and peat (*P* = 0.013, *P* = 0.04; Figure 3A). Chicks were more likely to be observed sleeping or resting in straw than in peat (*P* < 0.001), sand (*P* = 0.006), and pellets (*P* = 0.04; Figure 3B) and more likely in wood and hemp shavings than in peat (*P* = 0.006, *P* = 0.01; Figure 3B). Furthermore, chicks were more likely to be observed preening in straw and wood shavings than in peat (*P* = 0.003, *P* = 0.02; Figure 3C). Post hoc tests (pairwise signed-rank) show that birds were more likely to be observed dustbathing in sand than in hemp shavings (*P* = 0.02), straw (*P* = 0.02), and wood shavings (*P* = 0.04; Figure 3D), whereas peat showed a tendency of being more preferred than hemp, straw, and wood shavings (*P* ≤ 0.10; Figure 3D). Looking at the total number of observations of dustbathing birds, 41 occurred in sand, 26 occurred in peat, 3 occurred in pellets, and 1 occurred in wood shavings. No bird was ever observed to be dustbathing in hemp shavings or straw.

### Perch Type Preferences

**Figure 4:**
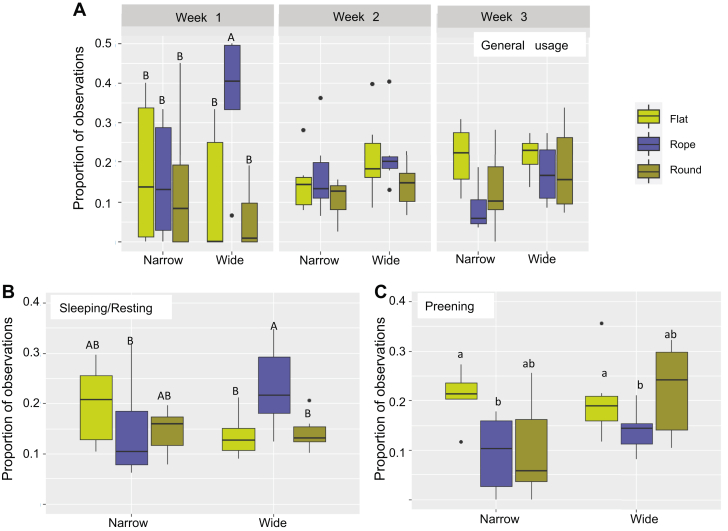
The boxplots show the average proportion of observations on the different types of perches (narrow or wide forms of the flat, rope or round perch shapes) (A) for birds observed on a perch irrespective of their behavior, but divided based on whether they were 1, 2, or 3 wk of age, (B) for birds observed sleeping or resting on a perch, and (C) for birds observed preening on a perch. ^A,B^ Different capital letters indicate significant (*P* ≤ 0.05) differences between perch types (both width and shape). ^a,b^ Different lower case letters indicate significant (*P* ≤ 0.05) general differences between perch shapes. Multiple pairwise comparisons have been controlled for using Tukey's method.

General perch use showed a significant interaction between perch type and week of age (F_4,90_ = 6.85, *P* = 0.02). Pairwise comparisons stratified on weeks showed that the wide rope was used more during the first week than any of the other perch types (*P* < 0.05), whereas no differences between perch types were found in the other weeks (Figure 4A).

The proportion of chicks sleeping or resting on a perch increased after the first week (week 1 = 0.10 ± 0.08, week 2 = 0.67 ± 0.08, week 3 = 0.68 ± 0.07; F_2,6.4_ = 21.26, *P* = 0.001), whereas sleeping and resting in litter decreased correspondingly (week 1 = 0.88 ± 0.07, week 2 = 0.33 ± 0.07, week 3 = 0.33 ± 0.07; F_2,6.5_ = 20.96, *P* = 0.001). However, there was no interaction between week and perch preference for sleeping or resting on a perch (*P* > 0.05). Instead, a significant interaction between perch width and shape was found for the average proportion of chicks sleeping on a perch (F_2,30_ = 3.52, *P* = 0.04; Figure 4B). Pairwise comparisons stratified on perch width showed that chicks slept or rested more on the wide rope than on the wide round (*P* = 0.03) and wide flat perches (*P* = 0.02; Figure 4B). Regarding the effect of width within perch shape, chicks slept or rested more on the wide rope than on the narrow rope (*P* = 0.04; Figure 4B).

Preening observations were found to be affected by perch shape (F_2,30_ = 4.65, *P* = 0.02; Figure 4C), wherein chicks were more likely to be observed preening on the flat perches than on the rope perches (0.22 ± 0.02 vs. 0.12 ± 0.02; *P* = 0.005; Figure 4C).

### Perch Balance

A total of 472 landing attempts on a perch were observed. Among these attempts, 120 were on the wide rope, 108 were on the narrow rope, 70 were on the wide flat, 68 were on the narrow flat, 65 were on the wide round, and 41 were on the narrow round. Of the total number of attempts, 295 resulted in a stable, “successful landing,” whereas in 177 landing attempts, birds showed balance movements or even failed to land on the perch, a “problematic landing.”

**Figure 5:**
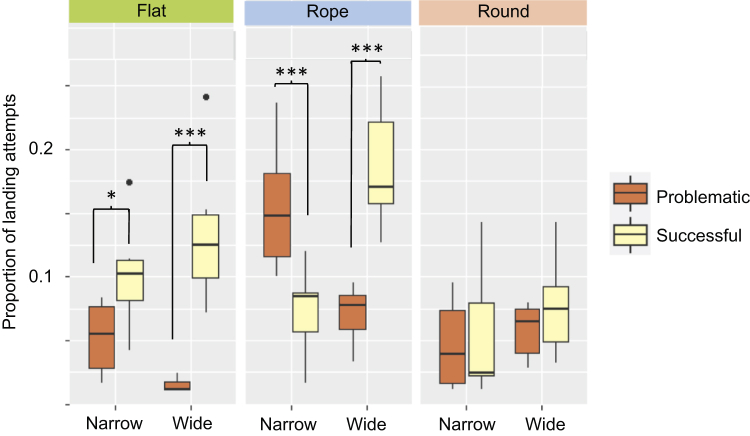
The boxplot shows the average proportion of a total of 472 landing attempts that were problematic or successful, on each of the 6 perch types; 2 widths (narrow and wide) and 3 shapes (flat, rope, and round). Asterisks indicate significant (*P* ≤ 0.05) differences between the 2 landing types within a perch type. Pairwise comparisons have been controlled for multiple comparisons using Tukey's method.

Regarding average proportion of landing attempts, a significant main effect of shape was found (F_2,55_ = 13.76, *P* ≤ 0.001). On average, birds tried to land more often on rope perches than on round (*P* ≤ 0.001) and flat perches (*P* = 0.003; Figure 5). In addition, an interaction between perch type and landing type was found (F_2,55_ = 7.65, *P* ≤ 0.001; Figure 5). Further investigations comparing landing type within each perch type showed that a landing attempt was more likely to be successful on the narrow flat (*P* = 0.05), the wide flat (*P* ≤ 0.001), and the wide rope perch (*P* ≤ 0.001; Figure 5) than it was to be a problematic landing. On the contrary, a landing attempt on the narrow rope perch was more likely to be problematic than it was to be successful (*P* ≤ 0.001). Attempting to land on the 2 round perch types was equally likely to result in a problematic as in a successful landing (*P* > 0.05; Figure 5).

## Discussion

Our study has shown that chicks use litter and perch types for different behaviors when given the option to choose. Chicks foraged mainly in wood shavings, hemp, and sand, and they dustbathed almost exclusively in sand and peat. They used the wide rope perch more often in their first week, and over the 3-week observation period, chicks used the wide rope perch most for sleeping or resting, whereas preening was most frequently observed on flat wooden perches. Furthermore, chicks were more likely to land successfully on the wide rope perch and the flat wooden ones. In summary, we conclude that giving chicks a choice of litter, which includes the option of either sand or peat, and a wide flat wooden perch and a wide braided rope perch (or a new perch type with their combined characteristics) would be appropriate to promote early use of these essential resources and thus help reduce some of the welfare problems commonly seen in adult laying hen flocks.

### Litter Preference

Chicks were observed to forage in around 50% of our observations, which corresponds to time budgets observed in semiwild relatives to the domestic fowl and red jungle fowl that forage around 60% of their active period (Dawkins, 1989). This suggests that the environment in our study could promote chicks' foraging behavior in similar ways to a natural surrounding. Chicks foraged least in peat and pellets. They foraged most in wood shavings, in hemp shavings, or in fine sand, even if hemp or sand was not more preferred for foraging than straw. These preferences are similar to those of Sanotra et al. (1995) who found that chicks preferred to ground peck in sand, straw, and wood shavings equally. Unlike our findings, Vestergaard and Baranyiova (1996) found lower levels of pecking and scratching in sand compared with peat in 2-week-old chicks. Research on preferences in adult laying hens has found peat to be similarly preferred to wood shavings and sand as a foraging substrate, when exploring usage in a push-door experiment setup (de Jong et al., 2007). It is not possible to say whether these slight differences reflect variation in the type of sand, the age of the birds, or the experimental setup. However, given the correlation between lower frequencies of foraging in the rearing environment and later severe feather pecking (Gilani et al., 2013; Rodenburg et al., 2013), this result suggests a higher likelihood of positive outcomes if presenting wood or hemp shavings, straw, or fine sand in the rearing environment for laying hen chicks because the higher preference could stimulate foraging behavior. However, Newberry et al. (2007) found that young individuals displaying higher levels of foraging behavior within a group were the same individuals showing an increased level of severe feather pecking as adults. This could further illustrate a common background for these behaviors. It could also support the importance of giving birds the opportunity to better fulfill their foraging needs, for example, by presenting litter types of varying characteristics, as birds in the study of Newberry et al. (2007) were only presented with wood shavings.

Our results show that chicks prefer different litter types for dustbathing than for foraging, similar to the results of the study by Sanotra et al., 1995, apart from sand being found to be preferred for both behaviors in our study. Our results indicate a preference for dustbathing in sand and peat and are as per earlier studies in young chicks (Sanotra et al., 1995; Shields et al., 2004). Chicks were only observed to dustbathe on one occasion in wood shavings and never in hemp shavings or straw, leading to the conclusion that these 3 materials are insufficient dustbathing substrates. Other studies have tried to train or promote dustbathing in wood shavings and straw, although sand (peat was not presented as an option) was still the preferred substrate even after the training period (Sanotra et al., 1995). If sand or peat is not provided in the early environment of laying hen chicks, it could, irrespective of later provision of these substrates, result in adults that are dustbathing less often (Johnsen et al., 1998) or performing insufficient dustbathing movements (Olsson et al., 2002), which may impair the hygienic function and thus risk poorer plumage condition (van Liere and Bokma, 1987). 10.13039/100014337Furthermore, low dustbathing activity when young can lead to severe feather pecking activity in adult age (Newberry et al., 2007), which further supports the importance of giving birds the possibility to fulfill behavioral needs also at younger ages.

It has been suggested that litter preference for dustbathing could be affected by substrate particle size and that small particles, such as those found in sand and peat, could be more preferred because of their better ability to get in between the feathers (van Liere and Bokma, 1987; Olsson and Keeling, 2005). The preferred foraging substrates, however, all had different particle sizes, suggesting some other characteristic to be of importance. A common feature could be color as the least preferred foraging substrates were the 2 darkest litter types tested. Chickens can discriminate colors as humans (Olsson et al., 2015), and it could be that particles in the light litter types are more visible to the birds and thereby promote foraging behavior better than the dark litter types.

Straw, a material not seen to be preferred for either foraging or dustbathing in our study or previous studies (Sanotra et al., 1995; Johnsen et al., 1998), was found to be the preferred substrate for resting together with wood and hemp shavings. Chicks were observed to be in straw for 30% of the observations when they were sleeping or resting in litter. Despite the increase in sleeping or resting on perches with age, chicks were observed resting twice as often in litter as on the perches during the first 3 wk and never observed sleeping or resting on the concrete. It is possible that the litter types most preferred for sleeping or resting (i.e., straw, hemp, and wood shavings) could all have better insulating properties, as previously reported for straw (Tuyttens, 2005), than the other substrates. The higher likelihood of observing preening in the same substrates preferred for resting and sleeping may indicate that chicks were relaxed and comfortable in these litter types (Spruijt et al., 1992).

In terms of implementation, certain litter types can have positive or negative effects on ammonia levels and on the concentration and production of dust in an aviary system (Gustafsson and von Wachenfelt, 2004), all of which can have large effects on laying hen health and welfare (David et al., 2015a, David et al., 2015b). Peat and straw can keep ammonia levels and total dust concentration low, but can lead to a high production of dust (mg/bird/hour) (Gustafsson and von Wachenfelt, 2004). Wood shavings can keep dust production and concentration low, but can be associated with high ammonia levels. Sand could lead to increased ammonia levels, dust production, and dust concentration and thereby seems unsuitable for production settings in this respect (David et al., 2015a, David et al., 2015b).

### Perch Preference and Perch Balance

We have shown that perch type, such as width and shape, can affect chicks' perch use, as has been previously shown in adult hens (Pickel et al., 2010; Chen et al., 2014; Scholz et al., 2014, among others).

The EFSA Panel in 2015 recommended, based on previous research on adult hens, that round perches could serve as the perch shape with highest usage and lowest risk of keel bone deformities. In addition, it is common to use round perches in multilevel systems for laying hens (EFSA, 2005; EFSA, 2015). Round perch types were not preferred over any other shape by the birds in our study, and birds were less likely to attempt to land on this perch shape. Instead, the wide rope perch was seen to be preferred, not only as the first perch to be used by young chicks but also for sleeping or resting. This could be explained by the higher likelihood of landing successfully, perhaps leading to greater security and thus further facilitating perch use. The earlier a chick starts using a perch, the more this individual uses perches for nighttime roosting in adult age (Heikkilä et al., 2006). This, in turn, can lead to lower fear levels, reduced aggression, and higher body condition scores in commercial laying hen flocks (Donaldson and O'Connell, 2012), all of which further support the possible positive outcome if presenting wide rope perches in the early rearing environment.

An overall effect of increased balance and use with wider perches, as found by Pickel et al. (2010), was somewhat supported by our results in chicks. The 2 widest perches were the 4.5-cm wide rope and 6.7-cm wide flat perch, both showing high usage and providing good balance (having more successful than problematic landings on them). However, birds showed better balance when landing on the 1.5-cm narrow flat perch (more successful landings than problematic) than when landing on the 3.5-cm wide round perch (as many problematic as successful landings), implying that the round shape negatively affected balance irrespective of width. Pickel et al. (2010) suggest balance movements to be the most sensitive indicator when identifying an appropriate perch type, and our results thereby support the use of flat perches for young chicks. That chicks were stable and thus perhaps felt safe on the flat perches could have explained the increased preening on this perch shape. The slight swinging of the rope perches when the bird was making preening movements could have led to a feeling of instability and thus less preening. The narrow rope perch, being both round and swinging slightly, seemed to result in a high proportion of problematic landings and low perch use in general.

The combination of the braided texture and breadth of the wide rope perch provided both possibilities for grip and a wide surface for the chicks to stand on, suggesting that these could be shape characteristics that promote early perch use. It could also be suggested that the wide rope was most similar to the wooden branches often used as perches under natural conditions. No wounds or damage was found on any bird during our study, but the potential risk of keel bone deformities and foot health for such a perch type is left to be explored in a more long-term study.

Regarding effects of materials, EFSA Panel on Animal Health and Animal Welfare, 2005, EFSA Panel on Animal Health and Animal Welfare, 2015 ranked rubber the highest, followed by wood and plastic. Perch shape in our study was dependent on perch material because wood was used for both our flat and round-shaped perch types. However, this was done with the aim of better exploring the effect of shape and width. Wood was chosen because it has been found to be a good material for perches based on usage (Appleby et al., 1992; Pickel et al., 2010; Chen et al., 2014), foot health, plumage condition (Appleby et al., 1992; Valkonen et al., 2005), and slipperiness (Scott and MacAngus, 2004). However, Scott and MacAngus (2004) suggested no difference between materials once perches are covered with manure. Rope may not be an appropriate material for large-scale commercial practice, from a hygiene perspective, but the braided shape that seemed to be preferred by birds in this study could potentially be formed in rubber.

### Including the Aspect of Choice in the Rearing Environment

The outcome of comparisons is always dependent, and a study is thereby limited by the number and variability of the types compared. However, the usefulness of results is increased when characteristics that resources have in common, such as particle size, color, shape or width, can be identified. We have shown the possibility and outcome when presenting different litter and perch types in the early rearing environment of laying chicks. That birds were seen to choose between these different types and prefer some over another in general, or for certain behaviors, contributes toward identifying the essential characteristics of the resources. But these results also highlight the potential of giving chicks choices to increase the likelihood of more chicks being able to appropriately perform motivated behaviors, such as ground pecking and perching, something that has been linked to lower levels of problems, such as feather pecking and floor eggs, in laying flocks. Giving choices is also an easy way to present an environment with increased possibilities to experience control, something that has been suggested to be crucial for an individual's well-being (Leotti et al., 2010).

## Conclusion

The first conclusion is that different behaviors were observed in different types of litter and that wood shavings, hemp shavings, fine sand, and straw are suitable for foraging, whereas fine sand and peat are suitable for dustbathing. Hence, providing more than one type of litter in the rearing environment for laying hen chicks could better fulfill different behavioral needs in commercial settings.

The second conclusion is that both the width and shape of a perch will impact usage and perch balance for young chicks. Flat wooden perches (e.g., 1.5- to 6.7-cm width) or a braided rope shape (e.g., 4.5 cm in diameter) could be adequate perch types for laying hen chicks to promote early perch use, increase general usage, and improve perch balance. All these could be beneficial for bird welfare in commercial aviary systems.
